# Visual Symptoms Outcomes in Cavernous Sinus Radiosurgery and a Systematic Review

**DOI:** 10.7759/cureus.23928

**Published:** 2022-04-07

**Authors:** Alejandra Moreira, Kaory C Barahona, Juliana Ramirez, Victor Caceros, Leonor Arce, Alejandro Blanco, Tatiana E Soto, Eduardo E Lovo

**Affiliations:** 1 Radiosurgery, International Cancer Center, Diagnostic Hospital, San Salvador, SLV; 2 Radiation Oncology, International Cancer Center, Diagnostic Hospital, San Salvador, SLV; 3 Radiosurgery, Centro de Radiocirugía Robótica, San José, CRI; 4 Radiation Oncology, Centro de Radiocirugía Robótica, San José, CRI

**Keywords:** visual outcomes, pituitary adenoma, meningiomas, cavernous sinus, radiosurgery

## Abstract

Introduction

The complex anatomy of the cavernous sinus confers a true challenge when it comes to safe tumor resection. Due to its non-invasive nature, stereotactic radiosurgery (SRS) is expected to have lower mortality and morbidity rates than microsurgery. The purpose of this study was to evaluate clinical results regarding visual symptoms after SRS for benign tumors invading the cavernous sinus. We also conducted a systematic literature review to provide a robust analysis regarding visual outcomes.

Methods

The study included 58 patients (43 women and 15 men; mean age: 52 years) with benign tumors invading the cavernous sinus (27 pituitary adenomas and 31 meningiomas) who underwent SRS with different platforms between August 2011 and December 2021. Of these, 26 patients underwent surgery before SRS, and the remaining 32 had SRS as first-line therapy. We identified symptoms involving cranial nerves (CN) II, III, IV, and VI in 38 patients at the time of SRS. We conducted a systematic review to identify all original studies assessing visual outcomes. We searched PubMed, the Latin American and Caribbean Health Sciences Literature index, and Google Scholar using the Medical Subject Heading search terms “radiosurgery” and “cavernous sinus” for valid studies published until January 31, 2022.

Results

Regarding pituitary adenomas, median tumor volume was 2.05 cc, 3.12 cc, and 2.39 cc for Gamma Knife (GK), CyberKnife (CK), and tomotherapy (Tomo), respectively. Median doses were 14 Gy for GK, 17 Gy for CK, and 15 Gy for Tomo. For meningiomas, median tumor volume was 10.2 cc, 2.62 cc, and 16.3 cc for GK, CK, and Tomo, respectively. The median dose was 14 Gy for GK, 14 Gy for CK, and 14.5 Gy for Tomo. The overall tumor control rate was 100% with a median follow-up of 33 months (range: 6-128 months). A reduction of >30% in total tumor size per the Response Evaluation Criteria in Solid Tumors (RECIST) classification was documented in seven patients (RECIST II; 12.1%), 51 patients (87.9%) had stable disease (RECIST III), and no increase in tumor volume was documented in any patient. Visual symptoms improved in 51.7% of patients. In the systematic review, the mean visual improvement was 36% (range: 25.8-42.5%).

Conclusion

SRS is an effective treatment for benign tumors invading the cavernous sinus. In this series, patients who underwent SRS as a primary treatment showed improvement in pre-existing cranial neuropathy and visual symptoms. Given the natural history of these tumors, which tend to grow and cause visual alternations, treating asymptomatic patients is a feasible approach worth considering for the appropriate patients.

## Introduction

The cavernous sinus is a paired venous structure located in the middle of the cranial base [1]. It is situated bilaterally to the sella turcica and extends from the superior orbital fissure anteriorly to the petrous part of the temporal bone posteriorly, and is approximately 1 cm wide and 2 cm long [2]. It contains a segment of the internal carotid artery (ICA), the sympathetic plexus, cranial nerves (CN) III, IV, and VI, and the ophthalmic (CN V1) and maxillary (CN V2) branches of the CN V [3].

Patients who suffer from tumors in the cavernous sinus commonly experience visual symptoms due to tumor infiltration. Numerous pathologies may involve the cavernous sinus; the most common are meningiomas, followed by pituitary adenomas, schwannomas, and hemangiomas [4]. There are no consensus guidelines on the management of cavernous sinus tumors; recommendations are based mainly on institutional practices, personal experiences, and the availability of treatment options.

Given the complex nature of cavernous sinus anatomy, treating tumors invading this area is of considerable interest to neurosurgeons; microsurgery in the cavernous sinus is challenging, as it contains vital neuronal and vascular structures elevating the risk of morbidity for surgical procedures [5-7].

Stereotactic radiosurgery (SRS) is a non-invasive treatment for benign tumors infiltrating the cavernous sinus, and it has shown to be effective in providing long-term tumor control. It can improve CN deficits and offers an adjuvant option for tumor residuals that show progression in this area. The proximity of these tumors to critical structures such as visual pathways requires special care to achieve optimal radiation doses to the tumor and avoid excessive exposure to organs at risk (especially the visual pathway). This study aimed to review the results of radiosurgery treatments of benign tumors involving the cavernous sinus with different radiosurgical technologies and compare visual symptoms outcomes reported in the literature via a systematic review.

## Materials and methods

We conducted a retrospective cohort study from August 2011 to December 2021 of all benign tumor cases involving the cavernous sinus. Patients were initially treated with tomotherapy (Tomo; Accuray Inc., Sunnyvale, CA, USA). In 2014, patients were treated with Infini, a rotating gamma-ray stereotactic system (MASEP Medical Science Technology Development Co., Shenzhen, China), and more recently, patients in a second center were treated with CyberKnife (CK; Accuray Inc., Sunnyvale, CA, USA). A total of 68 patients were identified, but 10 were excluded: five were lost to follow-up, four had malignant tumors, and one had less than six months of follow-up data. Therefore, 58 patients remained for this study.

All cases were reassessed with a particular interest in identifying tumor infiltration in the cavernous sinus, and all primary cases and recurrences were included. Variables such as sex, age, radiologic diagnostic, and symptoms prior to radiosurgery, primarily visual symptoms due to nerve compression, were considered among patients. Additionally, history of previous surgeries and the time from treatment to perceived radiosurgical effects regarding visual symptoms were also considered. During the last follow-up, radiological control was obtained among patients and graded according to the Response Evaluation Criteria in Solid Tumors (RECIST) classification [8]. All patients provided written informed consent to participate. The study was approved by the local Institutional Ethical Committee and Review Board.

Treatment planning

For Gamma Knife (GK), patients were fitted with a stereotactic frame after local anesthesia to the scalp. A T1-weighted multiplanar gradient recall gadolinium magnetic resonance imaging (MRI) with 1 mm slices was performed in all patients for treatment planning, and we conducted a 1 mm constructive interference in steady state (CISS) of the region of interest. Plans were reviewed to determine the tumor volume, dose delivered to the cavernous sinus, and the organs at risk, mainly the optic nerves, chiasm, and brainstem. For Tomo, patients were fitted with a PinPoint non-invasive frame (Aktina Medical, Congers, NY, USA) and underwent an MRI using the same sequences described above, and a computed tomography (CT) scan was performed on the day of simulation. Plans were performed using MIMVista software (MIM Software Inc., Cleveland, OH, USA). For CK, all patients experienced a simulation process, which consists of placing a thermoplastic mask to limit the patient’s head movements during treatment, and a contrast CT and T1-weighted multiplanar gradient recall gadolinium CISS MRI with 1 mm slices are then performed. Images are fused and plans are designed using MultiPlan (Accuray, Sunnyvale, CA, USA) and patients are treated 48-72 hours after treatment simulation. All patients treated with GK underwent a single treatment session, and those treated with Tomo or CK received either single or fractioned treatment sessions. All plans were reviewed and approved by neurosurgeons, radiation oncologists, and medical physicists.

Clinical observation and follow-up

Physical examination, including visual acuity and visual field testing, was performed on every patient prior to treatment, and additional reported symptoms were also considered. After treatment, follow-up was scheduled six months after radiosurgery and yearly after that; all patients have at least six months of follow-up data. Neuroimaging and visual controls were available for most patients to evaluate tumor response and visual symptoms improvement.

**Figure 1:**
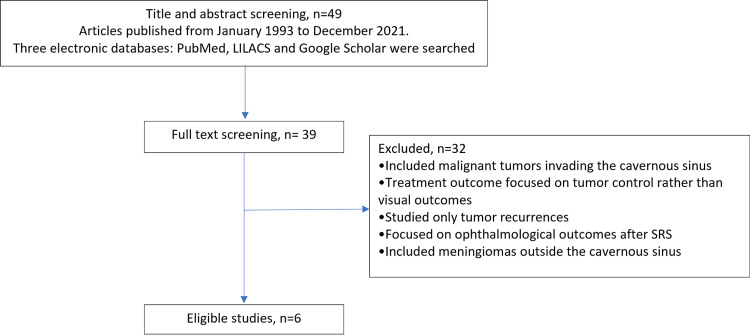
Scoping literature review LILACS, Latin American and Caribbean Health Sciences Literature; SRS, stereotactic radiosurgery.

To compare our results and determine the evidence of visual symptoms outcomes after SRS to the cavernous sinus, we conducted a scoping review to identify all original studies in which visual outcomes were assessed in the study. From database inception to January 31, 2022, we searched PubMed, the Latin American and Caribbean Health Sciences Literature index, and Google Scholar with Medical Subject Heading search terms “radiosurgery” and “cavernous sinus” (Figure 1).

## Results

Patient demographics are shown in Table 1. Of patients, 43 (74.1%) were females and 15 (25.9%) were males. The study population’s mean age was 52 years (range: 19-84 years), and the median follow-up was 33 months (range: 6-128 months). Two main tumors were identified among patients: pituitary adenomas and meningiomas. A total of 27 patients (46.6%) had pituitary adenomas and eight (29.6%) were secreting adenomas. A total of 31 (53.4%) patients had meningiomas. Surgery prior to SRS was documented in 26 (44.8%) patients; SRS was the initial treatment for 32 (55.2%) patients. Presenting symptoms were as follows: headache was present in three (5.2%) patients, 22 (37.9%) patients had CN II involvement, nine (15.5%) patients had CN III involvement, two (3.4%) patients had CN IV involvement, five (8.6%) patients had symptoms regarding CN V, five (8.6%) patients had CN VI involvement, and 13 (22.4%) patients were asymptomatic at the time of diagnosis. Of all patients presenting with visual symptoms, 19 (73%) went through a surgical procedure prior to SRS.

**Table 1:** Patient demographics CN, cranial nerve.

	Frequency, n (%)
Female	43 (74.1%)
Male	15 (25.9%)
Tumors	
Pituitary adenoma	27 (46.6%)
Meningioma	31 (53.4%)
Previous surgery	26 (44.8%)
Radiosurgery	
Infini	31 (53.4%)
CyberKnife	20 (34.5%)
Tomotherapy	7 (12.1%)
Presenting symptoms	
Headache	3 (5.2%)
CN II involvement	22 (37.9%)
CN III involvement	9 (15.5%)
CN IV involvement	2 (3.4%)
CN V involvement	4 (6.9%)
CN VI involvement	5 (8.6%)
Asymptomatic	13 (22.4%)

Table 2 shows the volume and dosimetry for each histology in this series. Regarding pituitary adenomas, median tumor volume was 2.05 cc, 3.12 cc, and 2.39 cc for GK, CK, and Tomo, respectively. Median prescription doses were 14 Gy for GK, 17 Gy for CK, and 15 Gy for Tomo. For meningiomas, median tumor volume was 10.2 cc, 2.62 cc, and 16.3 cc for GK, CK, and Tomo, respectively. The median dose was 14 Gy for GK, 14 Gy for CK, and 14.5 Gy for Tomo.

**Table 2:** Treatment planning for benign tumors GK, Gamma Knife; CK, CyberKnife.

Tumor	Radiosurgery (n)	Median tumor volume, cc (range)	Dose, Gy (range)	Isodose curve, % (range)
Pituitary adenoma	GK (10)	2.05 (1.5-17.1)	14 (12.5-18)	50% (49-55%)
	CK (12)	3.12 (0.146-11.8)	17 (13.3-25)	78% (75-83%)
	Tomo (5)	2.39 (1.27-40.2)	15 (13-22)	
Meningiomas	GK (21)	10.2 (1.0-33.3)	14 (11.5-20)	50% (50%)
	CK (8)	2.62 (0.98-18)	14 (13.3-21)	79.5% (75-83%)
	Tomo (2)	16.3 (5.3-27.2)	14.5 (13-16)	

Table 3 shows the overall symptom outcome after SRS. Of all patients, 13 who were initially asymptomatic (22.4%) remained asymptomatic after that, 36 patients (62.1%) reported symptoms improvement, seven (12.1%) had no change regarding their initial symptoms, and two (3.4%) had worsening symptoms (one patient with a CN III deficit and the other one with an increasing amount of painful trigeminal neuralgia). Particular emphasis was given to patients with visual symptoms involving CN II, CN III, CN IV, and CN VI. Of the 22 patients who presented with CN II involvement, five (22.7%) reported their symptoms improved after SRS without previous surgical treatment. Overall, visual symptom improvement was 51.7% for the whole series.

**Table 3:** Overall symptom outcome after SRS CN, cranial nerve; SRS, stereotactic radiosurgery.

Presenting symptoms	N (%)	Better, n (%)	Worse, n (%)	No change, n (%)
Asymptomatic	13 (22.4%)			13 (100%)
Headache	3 (5.2%)	3 (100%)		
CN II involvement	22 (37.9%)	19 (86.4%)		3 (13.6%)
CN III involvement	9 (15.5%)	5 (55.6%)	1 (11.1%)	3 (33.3%)
CN IV involvement	2 (3.4%)	2 (100%)		
CN V involvement	4 (6.9%)	3 (75%)	1 (25%)	
CN VI involvement	5 (8.6%)	4 (80%)		1 (20%)

Table 4 shows visual symptoms outcomes after SRS without previous surgery. Of all 58 patients, 18 (31%) had visual symptoms regarding CN II, CN III, CN IV, and CN VI without any history of previous surgery. Of those 18 patients, 14 (77.8%) reported progressive improvement of symptomatology after SRS, three patients (16.7%) had no change in their symptoms after radiosurgical treatment, and one patient (5.6%) reported worsening of symptoms (associated with CN III deficit). No further complications were documented among our patients after SRS.

**Table 4:** Visual outcomes after SRS without previous surgery CN, cranial nerve; SRS, stereotactic radiosurgery.

Visual symptoms	N (%)	Better, n (%)	Worse, n (%)	No change, n (%)
CN II involvement	4 (22%)	4 (100%)		
CN III involvement	7 (39%)	4 (57.1%)	1 (14.3%)	2 (28.65%)
CN IV involvement	2 (11%)	2 (100%)		
CN VI involvement	5 (28%)	4 (80%)		1 (20%)
Total	18 (100%)	14 (77.8%)	1 (5.6%)	3 (16.7%)

**Figure 2:**
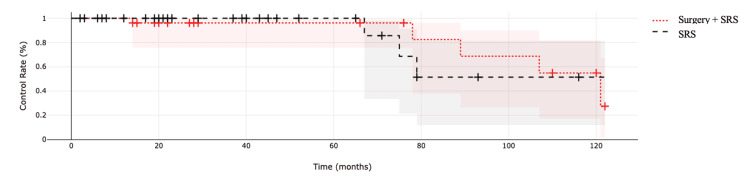
Tumor control rate at last follow-up in patients receiving surgery along with SRS vs. SRS alone SRS, stereotactic radiosurgery.

Regarding tumor control, seven (12.1%) patients had a reduction of >30% in total tumor size (RECIST II), and 51 (87.9%) had stable disease (RECIST III). Time to tumor progression was coded at the time of the first imaging study that showed tumor volume increase, but no increase in tumor volume was documented (RECIST IV). Within a median follow-up of 33 months (range: 6-128), symptom improvement ranged from 15 days to 180 days (six months), and the median recovery time was two months. Figure 2 shows the tumoral control rate.

For patients whose symptoms worsened, we further evaluated tumor volume, dose delivered to the cavernous sinus, and radiosurgical technology to determine if symptom deterioration was related to any of these factors. However, we found no statistical significance for any variable (tumor volume: p = 0.289; dose delivered: p = 0.235; radiosurgical technology: p = 0.123).

We identified 175 potential studies in the literature. A total of 49 studies were selected based on their title and abstract, and after a full-text evaluation of 39 records, only six studies showed information relevant to the current study (Table 5). One study demonstrated visual worsening; the rest showed a mean visual improvement of 36% (range: 25.8-42.5%).

**Table 5:** Study characteristics of published studies focusing on radiosurgery to the cavernous sinus and visual outcomes GK, Gamma Knife; CK, CyberKnife; Tomo, tomotherapy; SRS, stereotactic radiosurgery; ND, not documented; LINAC, linear accelerator.

Author (year)	Type of study	Sample size (N)	Radiosurgery technology	Dose range (Gy)	Overall tumor control (%)	Visual symptoms outcome improvement (%)	SRS as initial treatment (%)	Follow-up (months)
Tishler et al. (1993) [9]	Retrospective	62	GK; 6MV LINAC	8	ND	Reported complications rather than improvements	20%	19
Leber et al. (1998) [10]	Retrospective	50	GK	5-30	98%	25.8%	ND	40
Franzin et al. (2007) [11]	Retrospective	123	GK	10-20	98.4%	31.1%	66.7%	36
Hasegawa et al. (2007) [12]	Retrospective	115	GK	13	5 y: 87%, 10 y: 73%	46%	43%	62
Jensen et al. (2010) [13]	Retrospective	14	LINAC	15-20	79%	42.5%	ND	44.5
Kano et al. (2013) [14]	Retrospective	272	GK	13	3 y: 96%, 5 y: 94%, 10 y: 86%	1 y: 20%, 2 y: 34%, 3 y: 36%, 5 y: 39%	53.3%	62
Current series (2022)	Retrospective	58	GK, CK, Tomo	11-20 (GK), 14-25 (CK), 14-26 (Tomo)	100%	51.7%	31%	33

## Discussion

Patients who suffer from tumors in the cavernous sinus can experience visual symptoms due to tumor infiltration. Pituitary adenomas invade the cavernous sinus, sometimes encircling the ICA and extending to the temporal lobe. Meningiomas can originate within the cavernous sinus or can invade them while they arise from different surrounding areas such as the infraorbital, tuberculum sellae, sphenoidal crest, or petroclival areas. Meningiomas remain adherent to surrounding structures, bone, dura, sinuses, and the carotid wall, reducing the possibility of achieving complete surgical resection. Postoperative morbidity in cases of cavernous sinus tumors, particularly related to dysfunction of the CN, varies from 18% up to 63% [5,15-17].

One of the purposes of treatment for benign tumors is to effectively eliminate or control tumor growth, reduce symptoms, and avoid nerve injury, enabling patients a good quality of life [18]. The importance of radiosurgery in treating tumors in this area lies in its precision in delivering a high dose of radiation capable of controlling tumoral growth hopefully without damaging adjacent nerve functions [10]. Within the literature, there is a growing body of evidence showing the effectiveness of SRS in achieving long-term tumor control, but only a few authors have analyzed the effect of SRS on visual outcomes. CN radiation tolerance has been proposed since Tishler et al. [9] analyzed the damages resulting from radiosurgery to sensory and motor nerves passing through the CN and the optic nerve [9]. They found no complications when doses <25 Gy were delivered to CN III-VI. Contrarily, the optic nerves are allegedly more sensitive to radiation, thus keeping doses <8-10 Gy. Leber et al. and Morita et al. confirmed these observations with later reports [10,19]. Complications of healthy tissue after radiation have been known to correlate with dose, tissue sensitivity to radiation, and the volume irradiated [13]. Therefore, we sought to understand whether our cases that experienced neurological deterioration correlated with either dose, volume, or technology used. We did not demonstrate a relationship, but our series is relatively small, and further evaluation is needed.

Some studies focus on studying visual outcomes after SRS. Franzin et al., in a series of 123 patients and a median follow-up of 36 months, reported a 31.1% improvement in visual symptoms outcomes after SRS [11]. Most (66%) of their patients received SRS as a first treatment option, and GK was a useful treatment for cavernous sinus meningiomas as first-line or second-line therapy. GK was safe for tumors located near the optic pathways [11]. Hasegawa et al. evaluated long-term outcomes in patients with cavernous sinus meningiomas treated with SRS, reporting five-year and 10-year focal tumor control rates of 94% and 92%, respectively [12]. Regarding visual outcomes, 46% of their patients had some improvement, 43% remained stable, and 12% had worsening pre-existing symptoms. Also, 64% of their patients treated with SRS as initial treatment had symptom improvement, compared with only 34% of patients treated secondarily with SRS (p = 0.006). This suggests the possibility of permanent CN injury due to previous surgical resection [12]. Another study described a 37% improvement of pre-existing CN symptoms in patients who had undergone SRS as a first-line treatment, 44% showed improvement for oculomotor, 60% for trochlear, and 37% for abducens neuropathies in a five-year comparison [14].

The current study provides a series of patients with benign tumors invading the cavernous sinus initially treated with SRS; some presented with CN deficits, and others were asymptomatic. We had a median follow-up of 33 months. We report an overall 51.7% improvement in visual symptoms outcomes after SRS, and at the last follow-up, no patients suffered visual acuity loss or visual field defects. Additionally, 18 patients received SRS as their first-line treatment, of whom 14 (77.8%) experienced improved visual symptoms (CN II, III, IV, and VI), and to date, there have been no reports of subsequent CN deficit. From these patients at last follow-up, improvements were seen in 100% of patients with ocular neuropathy, 57.1% in those with oculomotor neuropathy, 100% in those with trochlear neuropathy, and 80% for those with abducens neuropathy. Those who underwent previous surgical resection had 61% improvement, and those who received SRS as a first treatment had 62.5% improvement. Our results are in accordance with those reported in the literature [11-14].

Two patients experienced worsening symptoms within our series: one patient with CN III deficit and one with an increasing amount of trigeminal neuralgia pain after SRS. The patient with the progressive CN III deficit had a long history of meningioma invading the cavernous sinus, had resigned surgical resection, and abandoned medical follow-up for several years; by the time of SRS, the patient’s visual symptoms were already affected. The patient with a meningioma invading the cavernous sinus experienced trigeminal pain relief after SRS, but the pain increased three months later. Several reports have documented an increase in trigeminal pain after treatment [9,20-22]. During the last follow-up, both patients had stable diseases (RECIST III).

Some patients within our series had an incidental finding of their tumor; 13 (22.4%) patients who were initially asymptomatic remained asymptomatic after SRS. The wider availability and increased use of neuroimaging for nonspecific symptomatology have increased the detection of incidental meningiomas [23]. Optimal management of asymptomatic, skull-based meningiomas is unclear and remains controversial [24-26]. Meningiomas arising primarily within the cavernous sinus are often believed more “silent” in their growth pattern, especially if they are not producing symptoms. At the same time, disabling symptoms can arise even with small tumors. Although there is existing literature regarding cavernous sinus meningiomas and their form of treatment, very little is known about their natural growth rate. Benjamin et al. studied the volumetric growth rates of untreated cavernous sinus meningiomas and found that the estimated volumetric growth rate was 23.3% per year, equivalent to a volume doubling time of 3.3 years and a median absolute growth of 41% within their patients [27]. They considered growth to increase greater than 20% during the observed period, and 65% of tumors demonstrated growth within their observational interval; a deeper understanding of the natural history of untreated cavernous sinus meningiomas allows for better management [28]. Additional studies focusing on the natural history of these tumors have reported radiological progression in linear diameters measurements to range between 39.5-45.7% and 39.5-48.1% in volumetric studies [23,29,30]. Studies reporting on the natural history of these tumors state that between 2.6% and 40% of patients with untreated, asymptomatic skull-base meningiomas will eventually experience symptoms requiring treatment [24]. A study compared the imagining and clinical outcomes of patients with asymptomatic, skull-based meningiomas managed with either SRS or active surveillance and found that SRS was associated with superior local tumor control (p = 0.001) compared to active surveillance, suggesting that if active surveillance is the initial means of management, SRS should be recommended when radiologic tumor progression is noted and prior to clinical progression [23].

Considering that 22.4% of our patients were initially asymptomatic and given the natural history of these tumors, which tend to grow and might cause visual deficits, in addition to the low morbidity rate in our series, treating asymptomatic patients is a reasonable approach.

There are several limitations to this study. First, it is a single-center experience with a retrospective design, which inherently limits its power and generalizability. Second, the overall patient population was small, limiting the possibility of a more complex statistical comparison. Follow-up duration was short, and therefore, cases of recurrence or symptoms progression over time were not completely identified and the literature reports visual improvement at five years. Prospective studies focusing on visual outcomes and longer follow-ups are still required.

## Conclusions

SRS is an effective treatment for benign tumors invading the cavernous sinus. In this series, most of the patients who underwent SRS as a primary treatment showed improvement in their pre-existing cranial neuropathy and visual symptoms. Given the natural history of these tumors and their tendency to cause visual alternations, treating asymptomatic patients with SRS might be a more reasonable approach than more conservative surveillance strategies.
